# Web-Based Physician Ratings for California Physicians on Probation

**DOI:** 10.2196/jmir.7488

**Published:** 2017-08-22

**Authors:** Gregory P Murphy, Mohannad A Awad, E Charles Osterberg, Thomas W Gaither, Thanabhudee Chumnarnsongkhroh, Samuel L Washington, Benjamin N Breyer

**Affiliations:** ^1^ Zuckerberg San Francisco General Hospital Department of Urology University of California, San Francisco San Francisco, CA United States; ^2^ Department of Surgery King Abdulaziz University Rabigh Saudi Arabia; ^3^ Dell Medical School Department of Surgery University of Texas Austin, TX United States; ^4^ School of Medicine Department of Urology University of California, San Francisco San Francisco, CA United States; ^5^ Department of Biostatistics and Epidemiology University of California, San Francisco San Francisco, CA United States

**Keywords:** online physician ratings, probation, Internet, quality of care

## Abstract

**Background:**

Web-based physician ratings systems are a popular tool to help patients evaluate physicians. Websites help patients find information regarding physician licensure, office hours, and disciplinary records along with ratings and reviews. Whether higher patient ratings are associated with higher quality of care is unclear.

**Objective:**

The aim of this study was to characterize the impact of physician probation on consumer ratings by comparing website ratings between doctors on probation against matched controls.

**Methods:**

A retrospective review of data from the Medical Board of California for physicians placed on probation from December 1989 to September 2015 was performed. Violations were categorized into nine types. Nonprobation controls were matched by zip code and specialty with probation cases in a 2:1 ratio using the California Department of Consumer Affairs website. Web-based reviews were recorded from vitals.com, healthgrades.com, and ratemds.com (ratings range from 1-5).

**Results:**

A total of 410 physicians were placed on probation for 866 violations. The mean (standard deviation [SD]) number of ratings per doctor was 5.2 (7.8) for cases and 4 (6.3) for controls (*P*=.003). The mean rating for physicians on probation was 3.7 (1.6) compared with 4.0 (1.0) for controls when all three rating websites were pooled (*P*<.001). Violations for medical documentation, incompetence, prescription negligence, and fraud were found to have statistically significant lower rating scores. Conversely, scores for professionalism, drugs or alcohol, crime, sexual misconduct, and personal illness were similar between cases and controls. In a univariate analysis, probation was found to be associated with lower rating, odds ratio=1.5 (95% CI 1.0-2.2). This association was not significant in a multivariate model when we included age and gender.

**Conclusions:**

Web-based physician ratings were lower for doctors on probation indicating that patients may perceive a difference. Despite these statistical findings, the absolute difference was quite small. Physician rating websites have utility but are imperfect proxies for competence. Further research on physician Web-based ratings is warranted to understand what they measure and how they are associated with quality.

## Introduction

Web-based physician ratings systems are becoming an increasingly popular tool to help patients choose their hospitals and providers [1]. Physician rating websites contain information regarding physician licensure, office hours, and disciplinary records in addition to ratings and reviews that are helpful to health care consumers [2]. Studies show mixed results as to whether highly rated hospitals or physicians deliver superior care [3,4]. Okike et al found no correlation between Web-based ratings for cardiac surgeons and 30-day mortality following coronary artery bypass grafting [5]. Regarding hospitals, a review of social media and rating site literature indicated a relationship between improved hospital ratings and better mortality and infection rates [6]. A systematic review by Doyle et al suggested a positive correlation between higher ratings and patient safety and clinical effectiveness [7]. Similarly, Greaves et al demonstrated that Web-based hospital ratings are associated with improved mortality and infection rates [8]. Higher Yelp ratings were also associated with higher Hospital Consumer Assessment of Healthcare Providers and Systems ratings [9]. Regarding social media, Facebook ratings have been correlated to lower hospital readmission rates, whereas Twitter comments were not associated with quality metrics [10,11].

When studying the quality of physician care, many different outcome metrics have been used with differing strengths. These outcomes can be influenced by a multitude of factors, some directly related to physician skill whereas others, such as patient health, are outside of the doctor’s control. Physician ratings have been correlated with such diverse outcomes as board certification, education, malpractice claims, mortality, infection, and readmission rates [2-12]. Though it can be difficult to equate physician competence with outcomes, doctors who violate codes of conduct and fail to meet the standard of care are placed on probation by their state medical board after a review of the evidence. It is unknown how patients perceive and rate physicians on probation as it has never been used as a quality metric. We sought to determine whether patients rated probated providers differently than physicians in good standing by comparing Web-based physician ratings from three consumer rating websites. We hypothesize that physicians on probation will have similar ratings to nonprobation controls.

## Methods

We retrospectively reviewed publically available data from the Medical Board of California for physicians who were placed on probation from December 1989 to September 2015. Rationales for probation were independently categorized into nine types of infractions by five independent reviewers (MAA, TWG, TC, SLW, and GPM). After reviewing all infractions, we used an inductive approach to create nine probation categories [13]. If there were any questions as to the appropriate categorization by an individual reviewer, this was brought to the group and a consensus decision was made. Nonprobation controls were matched by zip code and specialty with probation cases in a 2:1 ratio using the California Department of Consumer Affairs website. Web-based reviews were recorded from vitals.com, healthgrades.com, and ratemds.com. Ratings on these websites ranged from 1 to 5 and were weighted by the number of ratings so as not to overemphasize a small number of outlier ratings.

Statistical analysis was performed with STATA version 14 (College Station, TX). Parametric and nonparametric statistics were run despite the nonnormal statistical distribution. The results were similar between statistical analyses as the sample size was large [14]. For ease of reporting, parametric tests were reported. Specifically, a student’s *t*-test was used to compare mean ratings for violations. A rating below 3 was considered low and logistic univariable and multivariable regression analyses were done to determine predictors for low rating. We determined covariates including age, gender, type of specialty, and type of violation to be included in the multivariable model a priori. All tests were two-sided and *P* values <.05 were considered significant.

## Results

**Figure 1:**
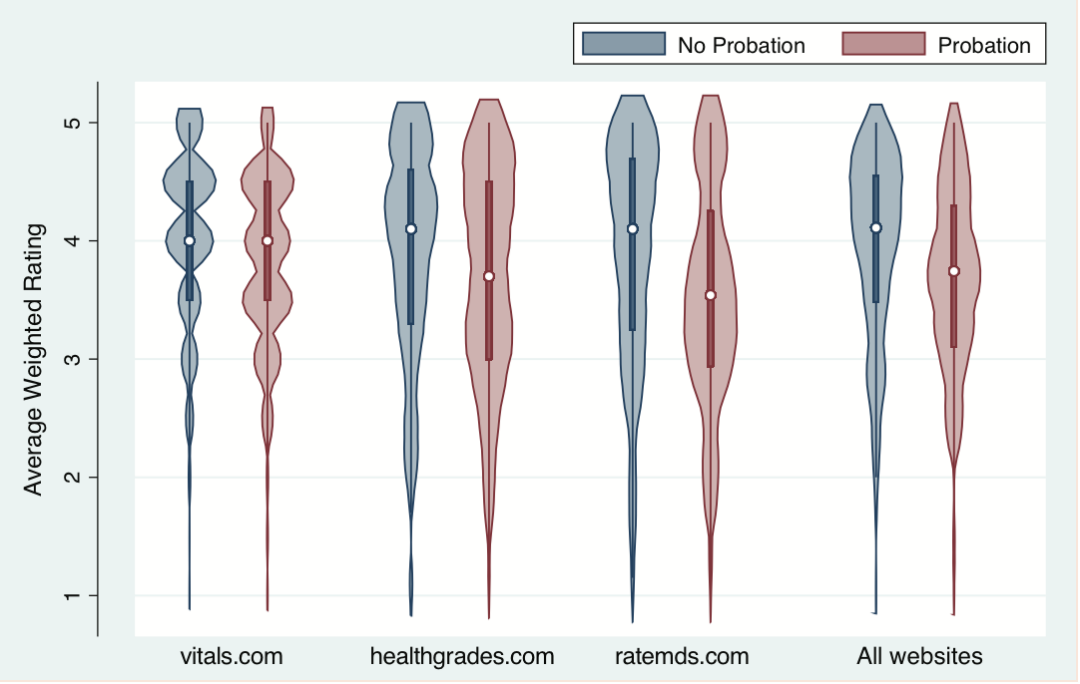
Violin plot showing ratings of physicians on probation versus controls by website.

A total of 410 physicians (cases) were placed on probation for 866 violations and were matched with 818 controls. The mean (standard deviation [SD]) number of ratings per physician was 5.2 (7.8) for cases and 4.0 (6.3) for controls (*P*=.003). The mean rating for physicians on probation was 3.7 (SD 1.6) compared with 4.0 (SD 1.0) for controls when all three rating websites were pooled (*P*<.001). Figure 1 depicts the overall violations using a violin plot that shows the median, interquartile range, and distribution of the ratings for cases and controls.

Tables 1 and 2 show the differences in average weighted ratings between cases and controls for each website stratified by violation type. Violations for medical documentation, incompetence, prescription negligence, and fraud were found to have statistically significant differences in physician ratings between cases and controls. Conversely, physician ratings for cases and controls were not statistically different when the violation pertained to professionalism, drugs or alcohol, crime, sexual misconduct, and personal illness.

**Table 1:** Mean rating of probation cases and controls by violation type.

Violation	Total (%)^a^	Vitals cases	Vitals controls	*P*-value	Health Grades cases	Health Grades controls	*P*-value
Medical records, mean (SD^b^)	176 (42.9)	3.7 (1.41)	4.1 (0.94)	<.001	3.6 (2.1)	3.8 (1.29)	.02
Professionalism, mean (SD)	166 (40.5)	3.9 (1.35)	4.1 (0.82)	.02	3.9 (1.98)	3.9 (1.09)	.81
Incompetence, mean (SD)	161 (39.3)	3.6 (1.46)	4 (0,93)	<.001	3.6 (2.08)	3.8 (1.21)	.08
Prescription negligence, mean (SD)	106 (25.9)	3.9 (1.6)	4 (1.05)	.31	3.8 (2.11)	3.9 (1.23)	.49
Drug or alcohol addiction, mean (SD)	84 (20.5)	4 (1.57)	4.1 (0.88)	.63	3.8 (2.1)	3.9 (1.11)	.66
Committing a crime, mean (SD)	63 (15.4)	3.8 (1.53)	3.8 (0.83)	.91	3.8 (1.87)	3.9 (0.94)	.45
Fraud, mean (SD)	44 (10.7)	3.9 (1.28)	4.1 (0.82)	.11	3.7 (1.81)	3.9 (1.05)	.32
Sexual misconduct or battery, mean (SD)	42 (10.2)	4 (1.27)	4.1 (0.81)	.28	4 (2.02)	4 (1.11)	.86
Personal illness, mean (SD)	24 (5.9)	3.7 (1.32)	4.2 (0.83)	.02	3.6 (2.3)	3.8 (1.09)	.56
All violations, mean (SD)	866 (0)	3.8 (1.47)	4.1 (0.96)	<.001	3.7 (2.03)	3.8 (1.2)	.01

^a^Some doctors have more than one violation. The percentage is for number of doctors on probation (cases group) having each violation out of the total number of doctors on probation (410).

^b^SD: standard deviation.

**Table 2:** Mean rating of probation cases and controls by violation type.

Violation	RateMDs cases	RateMDs controls	*P*-value	Cases in all websites	Controls in all websites	*P*-value
Medical records, mean (SD^a^)	3.5 (2.06)	3.8 (1.4)	.04	3.5 (1.58)	3.9 (1.04)	<.001
Professionalism, mean (SD)	3.9 (2)	4 (1.14)	.78	3.9 (1.58)	4 (0.87)	.54
Incompetence, mean (SD)	3.3 (2.21)	3.8 (1.43)	.03	3.5 (1.55)	4 (1.01)	<.001
Prescription negligence, mean (SD)	3.8 (2.12)	4 (1.32)	.41	3.8 (1.59)	4 (1.05)	.04
Drug or alcohol addiction, mean (SD)	3.8 (1.93)	3.9 (1.09)	.86	4 (1.78)	4 (0.93)	.64
Committing a crime, mean (SD)	3.7 (2.1)	3.7 (1.05)	.91	3.9 (1.59)	3.8 (0.83)	.62
Fraud, mean (SD)	3.3 (2)	3.6 (1.23)	.35	3.6 (1.37)	3.9 (0.89)	.03
Sexual misconduct or battery, mean (SD)	3.5 (1.83)	3.8 (1.11)	.26	3.9 (1.53)	4 (0.94)	.59
Personal illness, mean (SD)	3.4 (1.65)	3.4 (1.32)	.95	3.6 (1.33)	3.9 (0.8)	.17
All violations, mean (SD)	3.5 (2)	3.9 (1.31)	.001	3.7 (1.56)	4 (1.01)	<.001

^a^SD: standard deviation.

In the univariable analysis, probation was found to be associated with lower rating, odds ratio=1.5 (95% CI 1.001-2.2). This association was not significant in a multivariable model when we included age and gender, odds ratio=1.4 (95% CI 0.9-2.2). In addition, age, gender, type of specialty, and type of violation all did not predict a low rating.

Healthgrades.com included the probation status of the physician on their website in 328 of 389 (84%) cases in our cohort, yet there was no significant difference in mean ratings for doctors who had their violations published compared with those who did not. A sensitivity analysis was done to determine if timing of probation effected ratings. The majority of our violations are from the recent past. When we excluded violations before 2005, only 17 physicians were excluded and no difference in results was seen.

## Discussion

### Principal Findings

Web-based physician ratings from three websites were lower for doctors on probation, indicating that patients perceive a difference in physician performance. Despite this difference being statistically significant, the absolute difference is quite small with overlapping rating distributions. The vast majority of reviews were positive with small numbers of reviews per doctor. This is similar to other studies, making it difficult to draw strong conclusions about Web-based rating utility [6,15].

When categorizing the violations into subtypes, patients rate physicians lower when the probation infringements correspond to infractions for medical documentation, incompetence, prescription negligence, and fraud. In contrast, doctors on probation for professionalism, drugs or alcohol, sexual abuse, or personal illness received statistically similar ratings to controls. This finding may reflect what websites are measuring: patient perception and consumer experience, which is influenced mostly by bedside manner, wait times, and staffing issues [16]. Physicians with violations for medical documentation, for example, may run higher volume clinics, which negatively influences the patient experience. A German study showed higher physician ratings correlated to lower number of patients in the practice more than quality metrics [17].

### Comparison With Prior Work

The relationship between physician skills, Web-based ratings and outcome metrics is complex. Polled physicians are concerned about the usefulness of Web-based ratings and how they could negatively impact their practice [18]. Comparing websites scores of individual sports medicine doctors revealed a low degree of correlation between websites raising concerns about their reliability [19]. When reviews were correlated with scores, very high and very low ratings were correlated with the patient’s perception of physician skill and quality, which would seem difficult for patients to truly know [20]. Similarly, Web-based reviews of hand surgeons showed very positive reviews were related to perceived competence [21]. This raises concerns among physicians as to the accuracy and potential harm of negative reviews [6].

For providers, it is an uncomfortable position to be publically judged by patients, which could significantly damage a doctor’s reputation and practice. Some physicians have responded with indifference or disdain for these reviews [12,22]. Others have embraced these ratings and made an effort to alter their behavior and practice to improve patient experience and scores [21]. When selecting a surgeon Web-based, polled patients listed Web-based reviews as a minor factor with insurance, office location, and hospital reputation as more important [23]. It seems most patients understand the limitations of Web-based reviews.

There are ethical pitfalls of Web-based reviews due to the possible financial gain from improved referrals. Most reviews appear genuine but careful study revealed a few anonymous reviews may be from the physicians themselves in an attempt to falsely raise their ratings [2]. Better regulation may be needed to prevent abuses by providers including asking or paying patients for positive reviews [23]. Some concerns have been raised by doctors that competitors or unhappy employees could easily pose as a patient and post a negative review [24].

Despite the negatives, Web-based reviews are likely to increase in importance and patient utilization [1]. Lee suggests Web-based reviews help improve physician-patient relationship by increasing transparency and trust [25]. Not only does this help patient decision making but also provides the physician with feedback that can improve patient experience.

### Future Research

More research is needed to explore different physician quality metrics to see if there is an association with positive reviews. Determining more precisely what aspects of the health care system patient feedback can measure and improve is important. Prior studies explored many objective criteria such as mortality, infection, or readmission rates [4,6,7-10]. These are important metrics but do not necessarily reflect physician decision making. For example, a surgical site infection may occur despite excellent sterile technique and appropriate guidelines-based antibiotic practice. It raises questions as to whether physicians should be judged on outcomes or more on their decision making. Our study is the first to explore the link between Web-based reviews and probation status. Using probation as a proxy for clinical incompetence seems to be imperfect. When a physician is placed on probation, there are clearly issues to be remedied, but whether this negatively affects clinical care is difficult to determine. Whereas more research is needed to determine if probation is a reliable marker for clinical incompetence, it would seem to be a good indicator for poor physician decision making on some level. In addition, differing violations would seem to affect physician judgment and quality in diverse ways so lumping all probation cases has its limitations. More research is needed into which violations more severely affect physician quality.

### Limitations

Our study has limitations. The timing of probation could be problematic as violations from many years ago could correlate poorly with recent reviews. However, a sensitivity analysis excluding 17 cases before 2005 showed similar results. We studied patient reviews of physicians practicing in California, so these results may not be generalizable to reviews from other states. Future research should be directed toward other populations to confirm our findings.

### Conclusions

Web-based physician ratings were lower for doctors on probation indicating that patients may perceive a difference. Whereas statistically significant, the absolute difference was quite small. Physician rating websites have utility but are imperfect proxies for competence. Further research on physician Web-based ratings is warranted to understand what they measure and how they are associated with quality.

## Abbreviations

SD: standard deviation
